# Aesthetics sets patients ‘free’ to recover during hospitalization with a neurological disease. A qualitative study

**DOI:** 10.1080/17482631.2021.1992843

**Published:** 2021-11-08

**Authors:** Malene Beck, Eileen Engelke, Regner Birkelund, Bente Martinsen

**Affiliations:** 1 Department of Neurology,Zealand University Hospital, Roskilde, Denmark; 2 College of Health Professions, Lienhard School of Nursing, Pace Universityr, Teachers College, Columbia University, New York City, NY, USA; 3 Department of Regional Health Research, Institute of Health Service Research (IRS), University of Southern Denmark, Odense, Denmark; 4 Institute of Health, Department of Nursing Science, Aarhus University, Campus Emdrup, Copenhagen, NV, Denmark

**Keywords:** Hospital environment, sensory impression, aesthetics, phenomenology, van manen

## Abstract

**Background:**

Patients with neurological symptoms are particularly sensitive to the quality of the sensory impressions to which they are exposed to during hospitalization.

**Aim:**

To understand the meaning of aesthetic experiences to patients afflicted with neurological diseases during hospitalization on a neurological unit.

**Method:**

Fifteen patients were invited to “walk and talk” supplemented by semi-structured interviews conducted in newly established aesthetic tableaus within the neurology unit. Data analysis was inspired by the hermeneutic phenomenological methodology of van Manen.

**Result:**

The data analysis identified three overarching themes that unfolded in the patients’ experiences of a more aesthetic environment. The themes were: *1) A safe place to avoid noisiness, 2) An invitation to homey activities, 3) A thoughtful consideration for being ill.*

**Conclusion:**

Aesthetic elements can enable a thoughtful and needed consideration that withholds momentarily imaginative and hopeful experiences to patients in a vulnerable situation. Thus, aesthetics, together with peace and quietness, can set vulnerable patients free to retreat and recover from the symptoms of neurological diseases.

## Introduction

Characteristics of most neurological diseases is that it challenges humans on its senses. It is also evident that hospitalization to the neurological unit has existential impact on people, hence, patients can experience loneliness during their stay at the hospital (Beck, et al., 2016, 2018, 2019, 2020). Recent studies have shown how patients are becoming nomads lurking around to find breathing spaces when they were not offered a calm and familiar environment (Beck et al., 2020). However, the quality within introducing aesthetics as a supportive act to patients during hospitalization with neurological symptoms remains uncertain. Thus, wondering how an aesthetic experiment would be experienced by patients in the neurological unit paved the way to develop, test and evaluate an intervention in clinical practice.

## Background

### Neurological patients are challenged on their senses

The word “neurology” is originally Greek and defines ’nerve’ in combination with logic. In medical terms, patients suffering from a neurological disease are diagnosed with a variety of alterations within the nerve system’s structure, functions or congenital diseases as well as certain muscular diseases. Many of the diseases have a significant mortality; hence stroke (cerebral thrombosis and stroke) figures as the third leading cause of death after heart disease and cancer. This often leads to permanent mental or physical disabilities, also in younger age groups of patients (Feigin et al., 2017). The interface of the neurological diagnoses is extensive and affects humans on different levels; hence patients suffering from a neurological disease are often afflicted not only physically, but also experience challenges mentally, socially and/or financially (Feigin et al., 2017; Ganesh et al., 2017; Klinke et al., 2015; Low et al., 1999; O’Connell et al., 2020; Penner & Paul, 2017; Shaw, 2015; Simon et al., 2018; Tanner, 2001; Wolf et al., 2020). Examples of neurological diseases are migraines, Parkinson’s, epilepsy, strokes, tumours, and various types of dementia, such as Alzheimer’s disease, or other subcategories of diseases.

Characteristic of most neurological diseases is that it challenges humans on its senses (McGough et al., 2018). For example, neurological patients may be affected by their ability to have an overview (executive functions) and be unable to sort through the impressions they get from their surroundings (visuospatial functions). Patients with neurological symptoms may be quickly disturbed by external stimuli and therefore benefit from a calm and manageable environment (Applebaum et al., 2016; Digby & Bloomer, 2014; McGough et al., 2018). The consequences of neurological disease can be extensive and therefore require healthcare professionals to be aware of the importance of the sensory impression to best meet the patients’ needs and wishes (Beck et al., 2020). Scientifically, we know that hospitalized patients with neurological symptoms are particularly sensitive to sensory disturbances that may result in experiences of loneliness and discomfort or homeliness can occur (Beck et al., ,^2018^, ^2019^, ^2020^).

### Aesthetic as an ancient virtue

In western history, specifically in Greek antiquity, aesthetics was important to human life and in the way of living (Birkelund, 2013). Temples of health in Greek and Turkey, for instance, were built in exquisite terrain offering a view; sunny, airy, and beautiful buildings facilitated patients’ holistic treatment (Birkelund, (2017)). Nursing has a long tradition of focusing on environmental factors in relation to illness. Already in 1860 the founder of modern nursing, Florence Nightingale, effected many important changes in patient care by increasing focus on how aesthetics were important to patients when healing from sickness (Nightingale, (2007)). Today, it is difficult to document that Nightingales thoughts on the importance of aesthetics when being sick, influenced the well-being and survival of patients in her own time. However, much of what she advised in the mid-to late 1800s still holds true today (Fontaine et al., 2001). Most often remembered for her pioneering work in improving hospital sanitation, Nightingale was one of the first to address topics such as lighting, noise, and sensory stimulation in hospitals. A central tenet woven throughout Nightingales’ writings is the idea that only nature cures, and that nursing’s role is to put the patient in the best situation for nature to act (Dossey, 2000). However, as pointed out by Fontaine et al. (2001), extending this philosophy to the 21st century, includes making every effort to optimize the patient’s environment by reducing stimuli and enhancing factors that promote a sense of well-being, relaxation, and sleep. Achieving this goal, is as daunting a task now, as it was in Nightingale’s era, especially with the advent of advanced technology, a predominant feature of modern critical care units (Fontaine et al., 2001).

### Aesthetic in healthcare

Reseachers have focused on how aesthetics is closely related to creating a therapeutic environment (homelike, attractive) and identified that aesthetic is important in enhancing the hospital’s public image (Caspari et al., 2011). An aesthetic environment contributes to improved staff morale and patient care (Ibid.). Research has highlighted the need for stimulation in the physical hospital environment during hospitalization (Anåker et al., 2018, 2019; Rosbergen et al., 2017, 2019; Shannon et al., 2019). For example, Anåker et al. (2018, 2019) discloses how patients experience the physical environment at a newly built stroke unit as lonely and suggest to undertake activities systematically in order to encounter patient’s mental well-being during hospitalization. Other studies highlight how patients’ activity is impacted by the environment and points out the importance of creating activity within the physical environment, hence these are evaluated as significant in relation to patients experiencing being embedded in a enriched hospital environment (Rosbergen et al., 2017; Shannon et al., 2019). Other studies have shown how hospital environment plays a significant role on the patients’ and their family members’ overall satisfaction with the hospital experience (Harris et al., 2002). Further, Trochelman et al. (2012)investigates how already implemented evidence-based design features affect the patients’ satisfaction with the hospital environment. They conclude that within nursing, physical environment needs to be recognized as a major influence in care delivery that ultimately impacts patient safety, satisfaction and quality of care (Trochelman et al., 2012).

The notion of aesthetics, in particularly, the impact of art during hospitalization has been investigated empirically (Nielsen et al., Nielsen, 2017a,; Stine Louring Nielsen et al., 2017; Trevisani et al., 2010). The effect of art in the hospital has been assessed in relation to the patients’ feelings and emotions (Trevisani et al., 2010) and has the potential to influence patients mood and well-being (Moss, 2014; Ulrich et al., 2011; Zhang et al., 2019). According to Stine Louring Nielsen et al. (2017) the aesthetic value in art contributes to creating an atmosphere where patients feel safe, socialize, maintain a connection to the world outside the hospital and support their identity. Thus, the quality of aesthetics contributes to health outcomes by improving patient satisfaction (Stine Louring Nielsen et al., 2017,2017)

### Theory on aesthetics

Through the lenses of philosophical-phenomenology and in particular Løgstrup’s thinking (1976, 1983, 1997), this study will bring attention to the significance of aesthetics defined as “the sensory attuned impression” (Løgstrup, 1997). According to Løgstrup (1983), the attunement in our surroundings impacts our minds through our senses and in this way, gives nourishment to sovereign manifestations of life. However, the attunement can also be of such a nature as to give rise to the opposite of these expressions of life such as mistrust and hopelessness (K. E. Løgstrup, 1976).

### Introducing aesthetic tableaus—An intervention

This study was based on an intervention, called Hospitality that aimed at introducing aesthetic tableaus using visible technical means to provide a change of (physical) scenery in the neurology unit. Characteristics of the setting at the neurological unit is illuminated in Beck et al. (2020).

With the support of external grants, three outside agencies were contracted to introduce tableaus that would best support the health and well-being of hospitalized neurological patients. The hospital had no written strategy of hospital decoration or no “in house” hospital interior designers. To create a positive atmosphere the use of uplifting colours and screens for shielding was introduced within tableaus. Also, the use of positive distractions such as pictures with simple nature motives (e.g., a boat) aligning with the season of the year, was chosen to be a part of the tableaus. Within the tableaus the use of existing and new furniture, accessories and lamps, which would allow the patients more independent control over their environment, was prioritized. Realistic plastic flowers that aligned with the season of the year were also incorporated. The tableaus were introduced in December 2018 and included 1) Hallway area called “Café—Appetit for mind and body”, 2) An aisle between two wards called “The Garden Room”, and 3) A patient comfort room called the ‘Dwelling Room‘. Large posters were placed strategically in the unit, welcoming patients and relatives in the aesthetic environment. Following video shows the process: https://www.youtube.com/watch?v=SGdtJz6dA_E&t=75s

### Views from the tableaus

**Figure:**
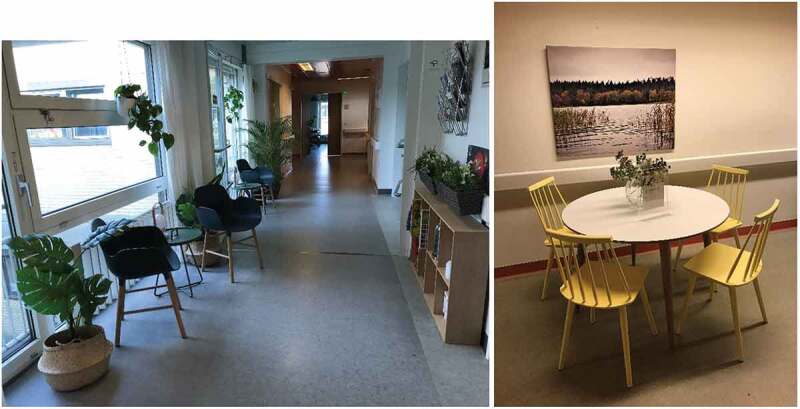

## The study

### Aim

To understand the meaning of aesthetic experiences to patients afflicted with neurological diseases during hospitalization on a neurological unit.

### Design

This study had an hermeneutic-phenomenological approach (van Manen, 2014), as we considered how aesthetic aspects would impact on the lived experiences of hospitalized patients afflicted by a neurological disease. Following the methodology of van Manen, 2014, a phenomenological descriptive sensitivity was combined with an interpretive understanding of the patients’ lived experience and how it was given meaning (van Manen, 2014). The methodology was well suited to explore day-to-day practice and to reveal unknown or sensitive aspects of the depths and subtleties of neurological patient’s experiences (ibid.).

### Participants

Fifteen patients were asked and agreed to be interviewed about the changed environment.

Taking into consideration that the participants were vulnerable and challenged on their senses, the first author spent some time at the unit from which the participants were recruited. This was to get acquainted with the setting and routines, trying to find the best possible time where it would be appropriate to ask the participants if they would be interested in participating in the study. To obtain information-rich data (Malterud, 2011), the participants were selected in collaboration with the staff, with special consideration to assure that chosen participants had used the aesthetic environment and were able to reflect on and express themselves about the meaningfulness of the newly established aesthetic tableaus in the neurological environment. Qualitative research strives for the greatest possible variation in the sampling of informants (D. Polit & Beck, 2018). A sample of 9 females, 6 males, age range (21–82) with a variety of severity of illness and length of stay were selected (Table I). The participants suffered from different disabilities e.g., dizziness, fatigue, physical impairment, pain, numbness, or sensitivity to light. The inclusion criteria included participants who spoke and understood (anonymous) and did not have other competing life-threatening disorders. Further, all participants should be adults (age 18+) and competent to provide experiences of the aesthetic environment. Exclusion criteria included participants with severe cognitive deficit or impressive/expressive aphasia or participants in an acute state of depressive suffering.

**Table I.** Overview of participants

No.	Gender	Age	Disease	Need of assistance	Length of stay
1	Male	42	Brain Tumour	1 person	5
2	Female	52	Brain Tumour	1 person	5
3	Male	78	Brain Tumour	1 person	2
4	Female	39	Epilepsy	None	3
5	Male	80	Unknown (Fainted in his home)	1 person	4
6	Female	52	Brain Tumour	1 person	4
7	Male	55	Brain Tumour	1 person	4
8	Female	67	Unknown (Numbness in her legs and dizzy)	None	4
9	Male	63	Cerebral haemorrhage	1 person	5
10	Female	52	Herpes Virus	None	4
11	Male	21	Unknown (Back-pain and numbness in right arm	1 person	4
12	Female	50	Alzheimer’s Disease	2 persons	2
13	Female	82	Unknown (Dizziness)	1 person	4
14	Female	39	Migraine	None	2
15	Female	73	Brain Tumour	1-person	5

## *Average in the unit was 3.5 days

### Data collection

To describe and verbalize what aesthetics are, how a sensory impression is experienced, as well as the significance of specific spaces (Birkelund, 2013; Van Manen, 2016) the first author performed “walk-along” interviews to capture the participants’ experiences of the aesthetic environment in the moment.

Researchers argue for the strengths of “walk-along” interviews, especially for research studies that will examine the importance of the environment in relation to health and well-being (Carpiano, 2009; Flick et al., 2019; King & Woodroffe, 2019; Kusenbach, 2006; Stiegler, 2020). Using “walk-along” interviews in this study (Carpiano, 2009; Flick et al., 2019; King & Woodroffe, 2019; Kusenbach, 2006; Stiegler, 2020) harmonized with the hermeneutic-phenomenological approach in which the participants’ immediate moments within the hospital environment, together with their reflections of the significance of aesthetic allowed us in coming to understand and explore parts of participants lifeworld (Van Manen, 2016). Thus, by exposing participants to the immediate, complex and subtle meaning of the aesthetic environment, the “walk-along” interviews addressed rich, nuanced and phenomenological sensitivity to the research question (Carpiano, 2009; Van Manen, 2017).

In practice, we gathered the empirical data by inviting the participants for a walk around the environment in which they were admitted. During the “walk-along” interviews, we explored themes related to their experiences. Neurological patients, due to their diagnosis (Parkinson’s, Alzheimer’s, stroke, etc.) may be physically challenged. For this reason, participants who had difficulty moving independently were offered a wheelchair during the “walk-along” interviews. Six (or however many) of the 15 patients in the study preferred (or required) utilizing the wheelchair over the walking. The “walk-along” interviews were addressed pedagogically by walking slowly and patiently, talking pauses, and listening carefully to the participants who spoked softly.

The “walk-along” interviews started with an open question e.g., “*show me a place in this unit that has made an impression on you?*” or “*Is there a certain place that you will like to show me in here?*” In order to gather the overall themes of the “walk-alongs” interviews and to reflect on the significance of the environment, all “walk-alongs’ ended in a supplementary interview. These interviews were conducted in one of the tableaus, sitting, and with focus on not being disturbed. A semi-structured interview inspired by Max van Manen’s theory of existential (van Manen, 2014; van Manen, 2014) was used. The guide contained suggested questions to help generate spontaneous and rich descriptions of the environment (Kvale, 2011b). The interviews were not “free” narration, but were structured with open-ended questions related to the environment impression (Ibid.).

The “walk-along” interviews were recorded with a small recorder, the size of a pen placed in the patient’s shirt, not inhibiting their movement in anyway. The length of the “walk-along” interviews varied and was in average 50 minutes.

### Ethics

Research among vulnerable participants may lead to ethical dilemmas and requires the researcher to be an ethical, knowledgeable and sensitive human being (Angel & Vatne, 2017; Kvale and Brinkmann, 2018). Therefore, the researchers in this study were guided by ethical principles to protect the study participants and ensure that the study was based on justice, beneficence and respect for human dignity (Damsgaard et al., 2020). The act of conducting “walk-along” interviews in this study was a moral practice wherein the interviewer was aware of the asymmetric relationship between the interviewer and the participant. Thus, to ensure that the participants felt confident to “walk-along”, attentiveness for the surrounds was imperative to assure safety for all (Angel, 2013). This attitude was achieved by creating a relaxed atmosphere using friendly and approachable body language (Fog, 2007). Senses and intuition were used when “walking” together with the participant to decide when to ask them to elaborate on their statements, to ask follow-up questions or to let silence and pauses take over (Angel, 2013). However, we were worried if walking interviews would be stressful for the participants given their neurological diseases. Therefore, we applied strategies easing conversation with people who live with cognitive and language impairment described by Kirkevold and Bergland (2007). e.g., the interviewer was special attentive to mimics, gestures and body language in general. Also unlimited time was allotted for the interviews attempting to create the best conditions for getting rich data despite the participants’ cognitive challenges. In the moment a participant showed any sign of exhaustion the interview was ended.

The study was performed in accordance with the ethical guidelines of the Nordic Nurses Federation and the Helsinki Declaration. Thus, written and verbal information about the study was given to all participants and informed consent was obtained. Participants were assured that their names and other personal information would be anonymized to maintain confidentiality. They were reassured that they could withdraw from the study at any time without any consequences for their treatment and care in the unit. According to (Anonymous) law, approval from the Regional Committee for Medical Research was not required because of the non-biomedical character of the study. The study was approved by the (Anonymous) Data Protection Agency, which requires safeguarding of any personal information and securing the anonymity of participants.

### Data analysis

The analytic steps in this study were guided by the thematic analysis described by van Manen, 2014. In practice, all interviews read several times, purposefully attending to embedded meanings during the “walk-along” interviews. Transcripts were read, in which the researchers searched for descriptions to answer the question, “*What is the experience of an aesthetic environments to patients afflicted with neurological diseases*?” The interview data was approached with an open-minded attitude about the real-life experiences embedded in the overall sense of “what was going on” (Van Manen, 2006; Van Manen, 1997). Afterwards, the text was clustered and analysed in order to identify understanding and meaning of d the material as a whole. The clusters were analysed and interpreted in the context of the overall understanding of the phenomenon by continuously going back and forth between clusters of meaning and the data material as a whole. Clusters were grouped into tentative themes to capture the phenomenon of interest (van Manen, 2014). These were presented to co-authors (EE & RB) to validate the preliminary interpretation and arguments for clustering (Van Manen, 2017).

During the analysis process, thematic statements were formulated as figures of meaning in concert with the above analytic reflective method to help point to possible eidetic meaning aspects of the phenomenon (van Manen, 2014). Eidetic refers to invariant patterns of meaning that may make a phenomenon distinct (van Manen, 2014). These thematic statements were used to structure the presentation of the text.

## Results

An overall finding was that the aesthetic environment was visible to the participants and an awareness of the quality of the physical environment made them thankful for the attempt to encounter their needs. This provided a sense of acknowledgement and belonging during their stay at the hospital. The participants interpreted the changes of the environment as a rewarding gesture that made the environment feel more patient-friendly. Despite the positive feedback of the aesthetic environment, participants expressed a distain for the disturbing noise they encountered during their stay. Un-fortunately, the noisy environment overshadowed the important calming potentials gained from the aesthetic environment. The data analysis identified three overarching themes that unfolded in the participants’ experiences of a more aesthetic environment. These themes shed light on how an improved physical aesthetic environment in the hospital is intertwined with disruptive noisiness. The themes are: *1) A safe place to avoid of noisiness, 2) An invitation to homey activities, 3) A thoughtful consideration for being ill.*

### A safe place to avoid noisiness

The participants described (an overwhelming sense of) how noisy sounds dominated the neurological setting. This meant that regardless any other (positive) sensory impression, the many distracting sounds from the environment “drowned out” the possible positive aesthetic experience that may have occurred because of the environmental changes. The participants explained that the noise within the neurological environment was both diverse and constant. Some of the examples of “noisy” activities were: people talking, visual and auditory tv activity, telephones ringing, sounds of cleaning machines, health professionals walking forth and back, other participants suffering e.g., moaning, screaming or crying, the sight of worn down furniture, movement of doctors’ rounds, physical training, and the persistent yet erratic sound of the calling system. The combination of these many activities provided a buzz, in which the participants experienced as transgressive and stressful, as it interfered with their experience of having peace of mind.

Well, the staff, they run back and forth. There is a lot of pace. ‘Please go in and talk quietly with the patients and ask how are you’ [A bell sounds] And THAT sounds right there. It’s special at night when someone needs help. It is very annoying and disturbing (P13). I can’t stand listening to the television, but it does increase the huge bangs coming from the hallway … and I do not get so scared when they [the staff] comes running in and makes quickly movements (P8).

The data identified that participants experienced their being in the neurological setting as challenging, and especially the noise they needed to escape from. The participants needed to process “something” in relation to their admission to the neurological unit. For example, for some participants it was challenging to encounter sensory impressions such as light, sound, or being able to concentrate. For others, one poor health report from the health professional made them worried or upset. These challenges potentiated the disruption that noise played in their recovery. The noise shaded the experience of being ill with comfortless and silenced the participants need for peace and quietness. A woman disclosed how this impaired code of conduct contributed to a less peaceful and pleasant environment. She felt the environment ideally should sound like:

There is no code in relation to peace and quietness in here. We talk on our phones and watch TV; the television … It’s just on all the time. Well, you might say, that there are no possibilities to be sick. Once, I experienced the alarm constantly ringing. It was loud and gave the feeling of ‘do I need to ring on that bell too or?’ It made me very confused (P9).

The participants described the aesthetic tableau as a way to escape the many noisy impressions that they dealt with in the hospital environment. In that sense, the established aesthetic tableaus in the neurological environment was a place where patients could avoid the *tumultuous* (p6) environment in the unit and the noisiness could step into the background, where patient needs were more in the foreground:

Here [the dwelling room] you can redraw to yourself. It’s a kind of a safe place. You are not completely gone from the everyday life … if a doctor should come around. You are comfortable at a distance … from the busyness (P2). I have sat down and looked at the two pictures [motives of a forest]. There’s a calmness in here … that means something because you can rest in any other places (P8) Here you can allow your imagination to take over; your mind can wander off. You go for a walk mentally (P4).

### An invitation to homey activities

In a neurological setting, recognizable furniture reminding one of a past time or of one’s home, generated an appreciated feeling of homeliness. The participants described how they used the “new living room” for homey activities such as talks with their significant others, drinking a cup of coffee, reading the newspaper, or to simply just sit and “be” for a while. Further, the room was used as a calm place to process new information about the course of their disease.

Yesterday I received bad news. I know that I asked for it, but nevertheless I got sad, and it is difficult to be sad among 4 fellow patients. So, my husband and I have been sitting in the Dwelling room, instead of escaping to the parking lot as we used to (P10).

Participants expressed that the nature pictures provided an opportunity to lose oneself in the rural scenes and “block out” the noisy surroundings. Enjoying the pictures and furniture often required that the participants be able to focus strictly on these elements, since hospital equipment (e.g., oxygen devices, walls filled with gloves, hand gel, or screens) was not experienced as inspiring at all:

I like them [pointing at the nature pictures with autumn colours]. They have beautiful colours and make me calm. If you look over there [on the opposite wall where respiratory devices are placed] … that is a whole different story. I try to avoid looking that way and concentrate on looking at the pictures instead (P7).

The “dwelling room” could also be experienced as welcoming change of scenery, where participants could escape the reality of the hospital and their disease:

One needs to change spaces. Yesterday, I was stressed because we had been sitting here [in the Dwelling room] talking until 9.45 p.m. and I thought that maybe they [the staff] had forgotten me and about my medication. I felt I had almost been away. That was a nice feeling (P14).

### A thoughtful consideration for being ill

The participants described ways in which the traditional environment was uncomfortable, such as the furniture being hard and unsuitable for ill people: *I noticed the chairs, they are wooden chairs; hard chairs. Ill people are not supposed to sit on chairs like these* (P1). Also, the lack of screen enclosures was repeatedly pointed to as a condition making it difficult to have privacy; both visual and auditory.

The aesthetic environment contrasted favourable with their traditional experience of the neurological environment. In the aesthetic tableaus a more patient-friendly consciousness towards the participants being ill was materialized within the decoration of the room. The participants explained how the environment was inviting them to sit down and relax. Here they had less sensory disturbances and the candles, lighting, and pictures with nature motives contributed to a calming atmosphere generating relaxation. A woman elaborates on how the aesthetic environment was a new place where she could recover:

So, when you need to recover, you need the television to be off … And no music, talk and stuff like that … Put simply, you should be able to relax and be calm when you are in the hospital. And here … [The ‘Café – Appetite for food and mind]) … it is just like that (P4).

The aesthetics of the tableaus made an overall positive impression on the participants. Several participants suggested that the interviews be conducted in the aesthetic environment, because they felt comfortable there. They described how the noisiness was better tolerated because the peaceful setting invited a peace of mind. In that sense, the interior was interpreted by the participants as having more than just a practical function, but it became a safe place, where they can “be ill” during their hospitalization. Being able to find peace of mind was particularly important to the participants since it promoted comfort and a needed moment to “collect” themselves during illness. A woman illuminates this by saying, while sitting in the “Dwelling-Room”:

I’ve been calmer now. And the pictures - they are beautiful. I have looked at the pictures. Trying to focus. I have trouble sleeping at night. So I went down here [dwelling room]. I just got more peace in here. In our patient room there was a lot going on. Someone who snores. Another is constantly checked by staff. Here, it is nice with all that stuff [pointing at the fake candles and on the pictures]. It’s about having something to look at. Just to calm down. That picture has some warmth and depth. That feeling is contagious (P3)

### Discussion

This study shows an overall understanding of what an aesthetic environment means to patients at the afflicted with neurological diseases. Thus, elaborates that aesthetics can reduce patient’s vulnerability during hospitalization by providing uplifting distraction that enables imaginative and hopeful experiences during hospitalization. In this study, participants shared their experiences of needing positively and thoughtful experiences to gain peace of mind. Such experiences provided calmness in a vulnerable situation: hence aesthetics could fill an existential void.

Nevertheless, our study also showed how an improved physical aesthetic environment in the hospital is intertwined with disruptive noisiness. Noisy sounds “owned” the neurological settings and participants needed to protect themselves and be shielding from clinical and disease-related sounds. Thus, the aesthetic places became concrete places in which the participants could distance themselves from fellow patients and avoid the inciting noise. In our recent study (Beck et al., 2020) we showed how hospitalized patients with neurological symptoms become nomads during hospitalization in order to find places to endure being present in the hospital environment. (Beck et al., 2020) paved the way for this recent study, in which we conducted an aesthetic experiment in clinical practice. This study provides new knowledge on how aesthetics, in terms of tableaus, are a helpful escape from the noisy environments in which patients wish to be protected from. However, our study also illuminates how an escape from the environment also have negative consequences for the sense of community with other people hospitalized with a neurological disease.

Our study illuminates how patients with neurological symptoms can be vulnerable to environmental stimuli. Purdy (2004) distinguishes between being vulnerable and being in a vulnerable situation. Purdy defines vulnerability as: A highly dynamic process of openness to circumstances that positively or negatively influence outcome (ibid.). This definition stresses that vulnerability is not created by the individual human being, but by the context in which the individual exists. Our study adds to an understanding of how environmental impression to patients depends on how health professional is able to handle aesthetics in the hospital context, hence ensuring sensory impressions not having a negatively impact on patients experiences of hospitalization.

Despite the impact of a noisy environment, our study showed positive effects of how the nature pictures and homey artefacts created an inner peace for the participants. Throughout history, there has been one quality that great leaders, policymakers, artists, and fighters have shared. Philosophers calls it “stillness”: the ability to be steady, focused and calm in a constantly busy world (Bollnow, 2011). In our study, the participants’ need for “stillness” was related to experiences that provided calmness and hope. The participants shared how the interpreted “stillness” was a momentary freedom from distress and worry. In a philosophical usage, the term “stillness” “a state of freedom from emotional disturbance and anxiety” (Ibid.). However, even though our study highlights the significance of aesthetics to patients, their experience of “stillness” was depending on how unnecessary tiresome sounds from devices or fellow patients was controlled or not controlled in the environment.

This study enhances that aesthetic elements may be experienced as a considerate homey invitation that offer imaginative moments where the inherent vulnerability can be encountered (Purdy, 2004). In other words, patients may benefit from aesthetics in the hospital environment, because it provides the experience of stillness to feed into a greater ambition to find relief within happy and peaceful moments during chronic sickness. However, noisiness holds the key to succeed in these aesthetic efforts. Thus, we recommend that existing hospitals are renovated under consideration to aesthetics and also that new hospitals are built with the same considerations together with a special attention to quiet tableaus. In this way the environment can set patients free to recover, and avoid that patients fading away in noisiness.

The evidence is clear. Noisiness has extensive consequences to ill people (Applebaum et al., 2016; Delaney et al., 2019; Garside et al., 2018; Laursen et al., 2014; Oleksy & Schlesinger, 2019). Our study supports previous studies on how a noisy environment is intertwined with a patient’s possibility to experience inner peace during hospitalization. The World Health Organization (WHO) has defined guidelines to sound environments in public space in order to be less harmful (Jarosińska et al., 2018). However, there is a lack of guidelines and systematic interventions of creating for peaceful hospital environments. This contrasts favourably to the current knowledge that hospitals are typically noisy, fast paced and create an overall disturbance to a person’s well-being (Beck et al., Beck, et al., 2016; Fillary et al., 2015; Konkani & Oakley, 2012).

### Strength and limitation

Within this study, trustworthiness was strived within the criteria of credibility, transferability, dependability, and confirmability (Lincoln & Guba, 1986; Nowell et al., 2017; Tobin & Begley, 2004). Credibility addresses the “fit” between respondents’ views and the researcher’s representation of them (Nowell et al., 2017). We conducted persistent data collection within the context of a neurology unit even though the design of “walk-alongs” was challenging. Further, we used international peer-debriefing in order to validate the result of the study. We tried to facilitate future transferability of our findings to other settings by using pictures from the unit and a careful description of the intervention. We kept records of raw data and conducted a reflexive journal during the study in order to systematize, relate and cross reference data. This helped ease the reporting of the research process and is aligned with the dependability criteria. Since, credibility, transferability and dependability were attained in this study; confirmability according to Guba and Lincoln and Guba (1986), was established. However, striving to fulfill the need for trustworthiness, this study had some limitations. One such limitation was that some of the participants lived with language impairments due to their neurological disease. These participants, however, contributed important and valuable experiences, yet not as “rich” as those who were able to truly articulate their experiences. Omitting these interviews would have given a narrower picture of the phenomenon under investigation (Kirkevold & Bergland, 2007).

We anticipated that the “walk-along” interviews would be an appropriate approach when asking participants about how the hospital environment to the patient was meaningful, and that discussions of the environmental factors influencing the participants “being” during hospitalization would be facilitated by indirect “talk as you walk” (Carpiano, 2009; King & Woodroffe, 2019; Stiegler, 2020). However, in practice, we experienced that in daytime, these walks were being interrupted, disturbed or cancelled by either health professionals wanting to do rounds, medication passes or other clinical staff eager to contribute to the investigation or care of the patient. Thus, seemingly the changes of the environment in this study were not comprehensive enough to counter the noise in the unit. Therefore, the full potential of the aesthetic environment may not be achieved. We then changed our walks to be conducted during evenings or weekends. This may have affected the data material, since the participants were interviewed in a calmer setting compared to the hectic dayshifts. The many noisy interruptions during data collection served as valuable illustrations and vivid data on the explicit need for quietness during hospitalization. In that sense, the methodological considerations was aligned with the purpose of the study (Polit & Beck, 2006, 2010; D. Polit & Beck, 2018; Whittemore & Melkus, 2008).

### Conclusion

This study sheds light on the importance of aesthetic elements within the hospital environment to patients in the neurological unit. Aesthetic elements have great impact on patients because they facilitate the experiences of being at home and safe, which are wanted by patients in their attempt to find relief during hospitalization. Our study focused on the quality of aesthetics, but did, however, identified how patients demanded peace and quietness within the environment to enjoy the impact of it. In this way peace and quietness emerged as a significant factor in how aesthetic elements (e.g., tableaus) can be experienced positively. Thus, aesthetic elements, together with peace and quietness, can set vulnerable patients free, which means that they can, to a greater extent, retreat and recover from neurological illnesses. Hence, aesthetic elements within the hospital environment focusing on silence can decrease further contextual vulnerability to patients with neurological diseases and in that sense encounter these patients’ needs for stillness.

### Relevance to clinical practice

The relevance of the study lies in its potential to inform hospital managers and staff members about how hospital environments play an important role in patient wellness and overall satisfaction of their care. It could be beneficial to patients afflicted with neurological diseases if health care professionals are determined to decrease to the level of noise in the wards. Our study may serve as a reminder of slowing down and harness the restorative wonders of serenity within the hospital walls. Furthermore, our study sheds light on the sustainable idea, that in order to move forward and develop clinical practice in a more patient-friendly way, clinicians could benefit from learning to be still in some ways during the everyday life at the hospital. Hence, stillness within the nursing discipline facilitates new ways of thinking and caring for persons while respecting their individual dignity and perspectives.

## References

[cit0001] Anåker, A., *et al*., (2019). “It’s Lonely”: Patients’ experiences of the physical environment at a newly built stroke unit. *Health Environments Research and Design Journal*. SAGE Publications Inc., 12(3), 141–11. 10.1177/1937586718806696 30336696 PMC6637812

[cit0002] Anåker, A., von Koch, L., Sjöstrand, C., Heylighen, A., and Elf, M., ., (2018). The physical environment and patients’ activities and care: A comparative case study at three newly built stroke units. *Journal of Advanced Nursing*. Blackwell Publishing Ltd, 74(8), 1919–11. 10.1111/jan.13690 29676493

[cit0003] Angel, S., & Vatne, S. (2017). Vulnerability in patients and nurses and the mutual vulnerability in the patient–nurse relationship. *Journal of Clinical Nursing*, 26(9–10), 1428–1437. 10.1111/jocn.13583 Blackwell Publishing Ltd 27626897

[cit0004] Angel, S. (2013). Grasping the experience of the other from an interview: Self-transposition in use. *International Journal of Qualitative Studies on Health and Well-being*, 8(1), 20634. 10.3402/qhw.v8i0.20634 no date 23972102 PMC3752432

[cit0005] Applebaum, D., Calo, O., & Neville, K. (2016). Implementation of quiet time for noise reduction on a medical-surgical unit. *The Journal of Nursing Administration*, 46(12), 669–674. 10.1097/NNA.0000000000000424 Lippincott Williams and Wilkins 27851709

[cit0006] Beck, M., Beck, M., Martinsen, B., Poulsen, I, and Birkelund, R ., (2016). Mealtimes in a neurological ward: A phenomenological-hermeneutic study. *Journal of Clinical Nursing*. Blackwell Publishing Ltd, 25(11–12), 1614–1623. 10.1111/jocn.13161 27094780

[cit0007] Beck, M., Birkelund, R., Poulsen, I., and Martinsen, B., ., (2019). Hospital meals are existential asylums to hospitalized people with a neurological disease: A phenomenological-hermeneutical explorative study of the meaningfulness of mealtimes. *Nursing Open*. Wiley-Blackwell Publishing Ltd, 6(2), 626–634. 10.1002/nop2.246 30918713 PMC6419126

[cit0008] Beck, M., Engelke, E, Birkelund, R., and Martinsen, B., . (2020). Becoming a nomad when hospitalized with a neurological disease: A phenomenological study. *International Journal of Qualitative Studies on Health and Well-being*, 15(1), 1815487. 10.1080/17482631.2020.1815487 32930071 PMC7534284

[cit0009] Beck, M., Poulsen, I., Martinsen, B., and Birkelund, R, ., (2018). ‘Longing for homeliness: Exploring mealtime experiences of patients suffering from a neurological disease. *Scandinavian Journal of Caring Sciences*. Blackwell Publishing Ltd, 32(1), 317–325. 10.1111/scs.12464 28840602

[cit0010] Birkelund, R (2017) Skonhed [Beauty]. In: Hospice: Æstetik, eksistens, og omsorg [Hospice: Aesthetics, eksistence, and care], (red.) Steenfeldt, V, O., Hansen, T. Munksgaard: Copenhagen.

[cit0011] Birkelund, R. (2013). Det æstetiske indtryks betydning for sundhed, sygdom og velvære Torsten, Risor. In *Sygdommens rum* (pp. 13–20). Tidsskrift for Forskning i Sygdom og Samfund.

[cit0012] Bollnow, O. F. (2011). *No Title* (8th ed. ; Edited by, T. Grifferro.). Mimesis International.

[cit0013] Carpiano, R. M. (2009). Come take a walk with me: The “Go-Along” interview as a novel method for studying the implications of place for health and well-being. *Health and Place*, 15(1), 263–272. 10.1016/j.healthplace.2008.05.003 18606557

[cit0014] Caspari, S., Eriksson, K., & Nåden, D. (2011). The importance of aesthetic surroundings: A study interviewing experts within different aesthetic fields. *Scandinavian Journal of Caring Sciences*, 25(1), 134–142. 10.1111/j.1471-6712.2010.00803.x 20626697

[cit0015] Damsgaard, J. B., Overgaard, C. L., & Birkelund, R. (2020). Personal recovery and depression, taking existential and social aspects into account: A struggle with institutional structures, loneliness and identity. *International Journal of Social Psychiatry*. SAGE Publications Ltd. 10.1177/0020764020938812 32611264

[cit0016] Delaney, L., Litton, E., & Van Haren, F. (2019). The effectiveness of noise interventions in the ICU. *Current Opinion in Anaesthesiology*, 32(2), 144–149. 10.1097/ACO.0000000000000708 Lippincott Williams and Wilkins 30817386

[cit0017] Digby, R., & Bloomer, M. J. (2014). People with dementia and the hospital environment: The view of patients and family carers. *International Journal of Older People Nursing*, 9(1), 34–43. 10.1111/opn.12014 Int J Older People Nurs 23320624

[cit0018] Dossey, B. M. (2000). *Florence nightingale: Mystic, visionary, healer*. Springhouse.

[cit0019] Feigin, V. L., and Vos, T., (2017). Global, regional, and national burden of neurological disorders during 1990–2015: A systematic analysis for the global burden of disease study 2015. *The Lancet Neurology*. Lancet Publishing Group, 16(11), 877–897. 10.1016/S1474-4422(17)30299-5 28931491 PMC5641502

[cit0020] Fillary, J., Chaplin, H., Chaplin, G., Show, J., and Wilson, P., (2015). Noise at night in hospital general wards: A mapping of the literature. *British Journal of Nursing*. MA Healthcare Ltd, 24(10), 536–540. 10.12968/bjon.2015.24.10.536 26018021

[cit0021] Flick, U., Hirseland, A., & Hans, B. (2019). Walking and talking integration: Triangulation of data from interviews and go-alongs for exploring immigrant welfare recipients’ sense(s) of belonging. *Qualitative Inquiry*, 25(8), 799–810. 10.1177/1077800418809515

[cit0022] Fog, J. (2007). *Det kvalitative forskningsinterview - med samtalen som udgangspunk*. Akademisk forlag.

[cit0023] Fontaine, D. K., Briggs, L. P., & Pope-Smith, B. (2001). Designing humanistic critical care environments. *Critical Care Nursing Quarterly*, 24(3), 21–34. 10.1097/00002727-200111000-00003 11858555

[cit0024] Ganesh, A., King-Shier, K., Manns, BJ., Hill, Michael, D., Campbell, David, JT., (2017). ‘Money is brain: Financial barriers and consequences for canadian stroke patients’, *Canadian Journal of Neurological Sciences*. *Cambridge University Press*, 44(2), 146–151. 10.1017/cjn.2016.411 27869051

[cit0025] Garside, J., Stephenson, J. Curtis, H. Morrell, M. Dearnley, C. Astin, F., (2018). Are noise reduction interventions effective in adult ward settings? A systematic review and meta analysis. *Applied Nursing Research*, 44, 6–17. 10.1016/j.apnr.2018.08.004 30389061

[cit0026] Harris, P. B. McBride, G. Ross, C. Curtis, L. (2002). ‘A place to heal: Environmental sources of satisfaction among hospital patients’, *Journal of Applied Social Psychology*. *Blackwell Publishing Ltd*, 32(6), 1276–1299. 10.1111/j.1559-1816.2002.tb01436.x

[cit0027] Jarosińska, D., and Héroux, ME, Wilkhu, P., Creswick, J., Verbeek, J., Wothge, J., Paunović, E.,(2018). ‘Development of the WHO environmental noise guidelines for the european region: An introduction’, *International Journal of Environmental Research and Public Health*. *MDPI AG*, 15, 4. 10.3390/ijerph15040813 PMC5923855 29677170

[cit0028] King, A. C., & Woodroffe, J. (2019). Walking interviews Pranee, Liamputtong. In *Handbook of Research Methods in Health Social Sciences* (SPRINGER) 1269–1290 . 10.1007/978-981-10-5251-4_28

[cit0029] Kirkevold, M., & Bergland, Å. (2007). The quality of qualitative data: Issues to consider when interviewing participants who have difficulties providing detailed accounts of their experiences. *International Journal of Qualitative Studies on Health and Well-being*, 2(2), 68–75. 10.1080/17482620701259273

[cit0030] Klinke, M. E. Klinke, M. Zahavi, D. Thorsteinsson, H. and Jónsdóttir, H. (2015). “Getting the left right” : The experience of hemispatial neglect after stroke.) 1623–1636. 10.1177/1049732314566328 25563629

[cit0031] Konkani, A., & Oakley, B. (2012). Noise in hospital intensive care units—a critical review of a critical topic. *Journal of Critical Care*, 27(5), 522.e1–522.e9. 10.1016/j.jcrc.2011.09.003 W.B. Saunders 22033048

[cit0032] Kusenbach, M. (2006). Patterns of neighboring: Practicing community in the parochial realm. *Symbolic Interaction*, 29(3), 279–306. 10.1525/si.2006.29.3.279

[cit0033] Kvale, S. (2011b). Enhancing interview quality. In *Doing interviews*. 10.4135/9781849208963.n12

[cit0034] Kvale, S, and Brinkmann, S. (2018). Doing Interviews. (2 ed.) Sage Publications Ltd: Copenhagen. 10.4135/9781849208963

[cit0035] Laursen, J. Danielsen, A., & Rosenberg, J. (2014). Effects of environmental design on patient outcome: A systematic review. *Health Environments Research and Design Journal*, 7(4), 108–119. 10.1177/193758671400700410 25303431

[cit0036] Lincoln, Y. S., & Guba, E. G. (1986). But is it rigorous? Trustworthiness and authenticity in naturalistic evaluation. *New Directions for Program Evaluation*, (1986)(30), 73–84. 10.1002/ev.1427 Wiley

[cit0037] Løgstrup, K. E. (1976). *Ophav og omgivelse – Betragtninger over historie og natur*. Nordisk Forlag.

[cit0038] Løgstrup, K. (1983). *Kunst og Erkendelse*. Gyldendal.

[cit0039] Løgstrup, K. (1997). *The ethical demand*., *Trans.* S. Hauerwas & A. MacIntyre. University of Notre Dame Press.

[cit0040] Low, J. T. S., Payne, S., & Roderick, P. (1999). The impact of stroke on informal carers: A literature review. *Social Science and Medicine*, 49(6), 711–725. 10.1016/S0277-9536(99)00194-X 10459884

[cit0041] Malterud, K. (2011). *Kvalitative metoder i medisinsk forskning : En innføring (qualitative methods in medical research: An introduction)*. Munksgaard.

[cit0042] McGough, N. N. H. Keane, T. Uppal, A. Dumlao, M. Rutherford, W. Kellogg, K. Ward, E. Kendal, C. Fields, W. (2018). Noise reduction in progressive care units. *Journal of Nursing Care Quality*. Lippincott Williams and Wilkins, 33(2), 166–172. 10.1097/NCQ.0000000000000275 28658188

[cit0043] Moss, H (2014). Perspectives The art of medicine Aesthetic deprivation in clinical settings. *Lancet*, 383, (9922), 1032–3. 10.1016/S0140-6736(14 24665475

[cit0044] Nielsen, S. L, Brorson, L. Roessler, K. Mullins, M. (2017a). How do patients actually experience and use art in hospitals? The significance of interaction: A user-oriented experimental case study. *International Journal of Qualitative Studies on Health and Well-being*, 12(1), 1267343. 10.1080/17482631.2016.1267343 28452607 PMC5328392

[cit0045] Nielsen, S. L, Mullins, M. Brorson, L. Roessler, K. (2017). The significance of certain elements in art for patients’ experience and use. *Visual Anthropology*, 30(4), 310–327. 10.1080/08949468.2017.1333360

[cit0046] Nightingale, F. (2007). (no date). *Notes on nursing: What it is and what it is not*. London.

[cit0047] Nowell, L. S., Norris, M. White, D. Moules, (2017). Thematic analysis: Striving to meet the trustworthiness criteria. *International Journal of Qualitative Methods*. SAGE Publications Inc., 16(1), 1. 10.1177/1609406917733847

[cit0048] O’Connell, N., Nicholson, T, R., Wessely, S., David, S. , ., (2020). ‘Characteristics of patients with motor functional neurological disorder in a large UK mental health service: A case-control study. *Psychological Medicine*. Cambridge University Press, 50(3), 446–455. 10.1017/S0033291719000266 30773149

[cit0049] Oleksy, A. J., & Schlesinger, J. J. (2019). What’s all that noise—Improving the hospital soundscape. *Journal of Clinical Monitoring and Computing*, 33(4), 557–562. 10.1007/s10877-018-0215-3 Springer Netherlands 30390171

[cit0050] Penner, I. K., & Paul, F. (2017). Fatigue as a symptom or comorbidity of neurological diseases. *Nature Reviews Neurology*, 13(11), 662–675. 10.1038/nrneurol.2017.117 Nature Publishing Group 29027539

[cit0051] Polit, D. F., & Beck, C. T. (2006). Essentials of nursing research: Methods, appraisal, and utilization. (6th ed.). Philadelphia, PA: Lippincott Williams & Wilkins. 10.1017/CBO9781107415324.004

[cit0052] Polit, D. F., & Beck, C. T. (2010). Generalization in quantitative and qualitative research: Myths and strategies. *International Journal of Nursing Studies*, 47(11). 10.1016/j.ijnurstu.2010.06.004 20598692

[cit0053] Polit, D., & Beck, C. (2018). Essentials of nursing research: Appraising evidence for nursing practice. In *BWolters Kluwer*.

[cit0054] Purdy, I. B. (2004). Vulnerable: A concept analysis. *Nursing Forum*, 39(4), 25–33. 10.1111/j.1744-6198.2004.tb00011.x Nurs Forum 15700483

[cit0055] Rosbergen, I. C. M., *et al*., (2019). The impact of environmental enrichment in an acute stroke unit on how and when patients undertake activities. *Clinical Rehabilitation*. SAGE Publications Ltd, 33(4), 784–795. 10.1177/0269215518820087 30582368

[cit0056] Rosbergen, I. C. M., Grimley, Rohan, S. Hayward, Kathryn, S. Walker, Katerina, C. Rowley, Donna. Campbell, Alana, M. McGufficke, Suzanna. Robertson, Samantha, S. Trinder, Janelle. Janssen, H. Brauer, Sandra, G. (2017). Embedding an enriched environment in an acute stroke unit increases activity in people with stroke: A controlled before-after pilot study. *Clinical Rehabilitation*. SAGE Publications Ltd, 31(11), 1516–1528. 10.1177/0269215517705181 28459184

[cit0057] Shannon, M. M. Swall, Anna. Anåker, Anna. Koch, Lena von. Elf, Marie. (2019). ‘Can the physical environment itself influence neurological patient activity? *Disability and Rehabilitation*. Taylor and Francis Ltd, 41(10), 1177–1189. 10.1080/09638288.2017.1423520 29343110

[cit0058] Shaw, G. (2015). Advocacy. *Neurology Now*, 11(2), 13. 10.1097/01.NNN.0000464320.10147.75

[cit0059] Simon, F., Barnay, J., & Lannuzel, A. (2018). The wide spectrum of neurological consequences of chikungunya disease. *Reviews in Medical Virology*, 6, e1999–n/a. 10.1002/rmv.1999 30074289

[cit0060] Stiegler, S. (2020). On doing go-along interviews: Toward sensuous analyses of everyday experiences. *Qualitative Inquiry* 27 (3–4) 364–373 . 10.1177/1077800420918891

[cit0061] Tanner, D. (2001). ‘Sustaining the self in later life: Supporting older people in the community. *Ageing and Society*, 21(3), 255–278. 10.1017/S0144686X01008248 Cambridge University Press

[cit0062] Tobin, G. A., & Begley, C. M. (2004). Methodological rigour within a qualitative framework. *Journal of Advanced Nursing*, 48(4), 388–396. 10.1111/j.1365-2648.2004.03207.x John Wiley & Sons, Ltd 15500533

[cit0063] Trevisani, F. Casadio, R., Romagnoli, F., Maria Zamagni, P., Francesconi, C., Tromellini, A., Di Micoli, A., Frigerio, A., Farinelli, G., Bernardi, M. , (2010). ‘Art in the hospital: Its impact on the feelings and emotional state of patients admitted to an internal medicine unit’, *Journal of Alternative and Complementary Medicine*. *J Altern Complement Med*, 16(8), 853–859. 10.1089/acm.2009.0490 20653484

[cit0064] Trochelman, K. Albert, Nancy., Spence, J., Murray, T., Slifcak, E. , . (2012). Patients and their families weigh in on evidence-based hospital design. *Critical Care Nurse*, 32(1), 1. 10.4037/ccn2012785 22298725

[cit0065] Ulrich, R. S., Ulrich, R. S., Berry, L. L., Quan, X., Parish, J. T., ., (2011). A conceptual framework for the domain of evidence-based design. *Health Environments Research and Design Journal*. Vendome Group LLC, 4(1), 95–114. 10.1177/193758671000400107 21162431

[cit0066] Van Manen, M. (1997). From meaning to method. *Qualitative Health Research*, 7(3), 345–369. 10.1177/104973239700700303 SAGE Publications Inc.

[cit0067] van Manen, M. (2006). Writing qualitatively, or the demands of writing. *Qualitative Health Research*, 16(5), 713–722. 10.1177/1049732306286911 Sage PublicationsSage CA: Thousand Oaks, CA 16611974

[cit0068] van Manen, M 2014 . no date *Researching lived experience : Human science for an action sensitive pedagogy*. 2 Ont: Althouse Press

[cit0069] van Manen, M. (2016). Writing in the dark: Phenomenological studies in interpretive inquiry. *Writing in the Dark: Phenomenological Studies in Interpretive Inquiry* (1. ed.) Routledge: New York. pp. 7- 268 . 10.4324/9781315415574

[cit0070] Van Manen, M. (2017). Phenomenology in Its Original Sense. *Qualitative Health Research*, 27(6), 810–825. 10.1177/1049732317699381 28682720

[cit0071] vanManen, M (2014) .no date. *Phenomenology of practice : Meaning-giving methods in phenomenological research and writing* (M. van Manen, Edited by).Taylor and Francis (Developing Qualitative Inquiry.).

[cit0072] Whittemore, R., & Melkus, G. (2008). Design decisions in research e-source - Behavioural & social sciences research. *The Diabetes Educator*, *34*(2), 201-16 . 10.1177/0145721708315678 18375773

[cit0073] Wolf, J., *et al*. (2020). From illness perceptions to illness reality? Perceived consequences and emotional representations relate to handicap in patients with vertigo and dizziness. *Journal of Psychosomatic Research*. Elsevier Inc., 130, 109934. 10.1016/j.jpsychores.2020.109934 31972479

[cit0074] Zhang, Y., Tzortzopoulos, P., & Kagioglou, M. (2019). Healing built-environment effects on health outcomes: Environment–occupant–health framework. *Building Research and Information*, 47(6), 747–766. 10.1080/09613218.2017.1411130 Routledge

